# Prevalence of postoperative complications in oncologic gastro-esophageal surgeries: a cross-sectional study

**DOI:** 10.1590/acb394424

**Published:** 2024-07-22

**Authors:** Laura Mota Vieira Lima, Paula Costa Guimarães, Daniele de Oliveira Montenegro, Fernanda de Sousa Filgueira, José Gomes, Ricardo Ney Cobucci, Kleyton Santos de Medeiros, Irami Araújo-Filho

**Affiliations:** 1 Liga Contra o Câncer – Natal (RN) – Brazil.; 2 Universidade Potiguar – Medical School – Health School – Natal (RN) – Brazil.; 3 Universidade Federal do Rio Grande do Norte – Postgraduate Program in Health Sciences – Natal (RN) – Brazil.; 4 Universidade Federal do Rio Grande do Norte – Department of Surgery – Natal (RN) – Brazil.

**Keywords:** Postoperative Complications, Surgical Oncology, Stomach Neoplasms, Esophagectomy, Gastrectomy

## Abstract

**Purpose:**

This study evaluated the prevalence of complications in the postoperative period of esophagogastric oncological surgeries.

**Methods:**

We conducted a retrospective cross-sectional study, adhering to the Strengthening the Reporting of Observational Studies in Epidemiology (STROBE) guidelines. The study size implied 163 patients who underwent surgical treatment for esophageal and gastric cancer and experienced postoperative complications between January 2018 and December 2022. These patients were treated at the Liga Norte Riograndense Contra o Câncer, a high-complexity oncology center and a reference for cancer treatment in Northeast Brazil.

**Results:**

The prevalence found was 88.3%. The most prevalent complications were Clavien-Dindo I and II, and infection was the most common. According to our statistics analysis, hypoalbuminemia showed a positive correspondence with the occurrence of postoperative complications (odds ratio = 8.60; 95% confidence interval 1.35–54.64, p = 0.0358).

**Conclusions:**

Postoperative complications of gastroesophageal surgeries increase patient morbidity and mortality.

## Introduction

The fifth most common cancer is gastric cancer, responsible for over a million new cases in 2018 and an estimated one in every 12 deaths globally, making it the second leading cause of cancer-related death^1^^–^ 3. A pathology of such relevance and aggressiveness demands treatments that require a high degree of surgical expertise. Despite the development of adjuvant treatment methods, total gastrectomy associated with the removal of affected lymph nodes remains the primary form of management.

Specifically for patients with gastric cancer, the occurrence of complications has a significant impact on reducing overall survival and disease-free survival^4^. Thus, the surgical outcome depends on the patient’s clinical condition, the type of surgery, and the quality of care. The multifactorial nature of this process predisposes the occurrence of surgical complications, which can be assessed by the Clavien-Dindo Classification of Surgical Complications. This classification, revised in 2004, consists of five severity grades^5^^–^ 7.

The Clavien-Dindo Classification of Surgical Complications ranges from grade I to V:

- Grade I: any deviation from normal postoperative conditions;
- Grade II: the use of clinical treatment with antibiotics or parenteral therapy;
- Grade III: divided into A and B, encompasses invasive interventions requiring or not general anesthesia;
- Grade IV: divided into A and B, includes severe patients requiring intensive care with single or multiple organ dysfunction;
- Grade V: death^5^^–^ 7.

According to this classification, several studies describe the main complications of surgical treatment, such as anastomotic fistulas, pulmonary dysfunction, and pain^8^. A mortality rate of 43% is also reported in patients undergoing esophagogastrectomy with extended lymphadenectomy^9^. Recognizing complications allows for the early identification of risk factors contributing to clinical outcomes and thus minimizes morbidity and mortality in the postoperative period for cancer patients^4^.

It is important to analyze local factors that influence the incidence of unfavorable postoperative outcomes to create strategies for prevention and optimized treatment^10^. Therefore, this study evaluated the prevalence of complications in the postoperative period of esophagogastric oncological surgeries.

## Methods

### Study design

We conducted a retrospective cross-sectional study, adhering to the Strengthening the Reporting of Observational Studies in Epidemiology (STROBE) guidelines^11^. The study size implied 163 patients who underwent surgical treatment for esophageal and gastric cancer and experienced postoperative complications between January 2018 and December 2022. These patients were treated at the Liga Norte Riograndense Contra o Câncer, a high-complexity oncology center and a reference for cancer treatment in Northeast Brazil, as well as an initiation and research center where this study was carried out.

### Patients

Data were obtained through the evaluation of electronic medical records and prospectively maintained in the patient database. The selected patients for the study met the following criteria: adults who underwent esophagectomy, esophagogastrectomy, or gastrectomy; with the esophagus, stomach, or esophagogastric junction as the primary histological site of cancer; and with sufficient information available for analysis.

### Demographic and clinical variables

Sociodemographic variables collected for the patients included age, biological sex (male and female), disease profile, treatment received, and recorded complications. The disease profile encompassed tumor location and histological subtype. Surgical modalities consisted of esophagogastrectomy, esophagectomy, and gastrectomy. Regarding the treatment profile, the type of surgery was recorded, and postoperative complications were documented according to the Clavien-Dindo Classification of Surgical Complications, considering complications within 30 days after the procedure.

The updated patient database also included the following variables: American Society of Anesthesiologists (ASA) score for determining surgical risk; whether the method was open or laparoscopic; procedure time less than 179 minutes, greater than 180 minutes, or greater than 360 minutes; whether there was more than one procedure during surgery; length of hospital stay; and whether there were reentries during this period.

Additionally, the use of antibiotic prophylaxis was considered only when administered 24 hours before up to 24 hours after the procedure. Preoperative nutritional assessment was considered up to 24 hours before the procedure. Preoperative laboratory tests (albumin level, hemoglobin, hematocrit, leukogram, and lymphocytes) were considered up to three months before the procedure. Anemic patients were defined as those with hemoglobin < 11 or hematocrit < 35%, hypoalbuminemia with albumin < 3.5, leukopenia for leukocytes < 4,000, and lymphopenia for lymphocytes < 900.

### Statistical analysis

The descriptive analysis is presented in tables using frequencies and their respective percentages. Non-parametric tests, including the χ^2^ test and Fisher’s exact test, were employed to assess the existence of associations between variables. Additionally, to identify potential factors associated with the studied outcomes, a logistic regression model was adjusted, and the odds ratio along with their respective confidence intervals were obtained. The significance level considered was 5%. The mortality rate was also calculated for patients undergoing gastroesophageal oncologic surgery.

Data were collected from patient records and stored using the Research Electronic Data Capture (REDCap) electronic data capture tool. REDCap is a secure, web-based software platform designed to support data capture for research studies. Data export was performed through the Microsoft Excel spreadsheet editor. Statistical analysis was conducted using the R programming language.

### Ethics

This study was conducted following the ethical principles of the Declaration of Helsinki. The study protocols were approved by the Research Ethics Committee of Liga Contra o Câncer, with the Certificate of Presentation for Ethical Consideration No. 65450522.3.0000.5293, Opinion No. 5.800.302/2022, issued on December 7, 2022.

## Results

**Figure 1:**
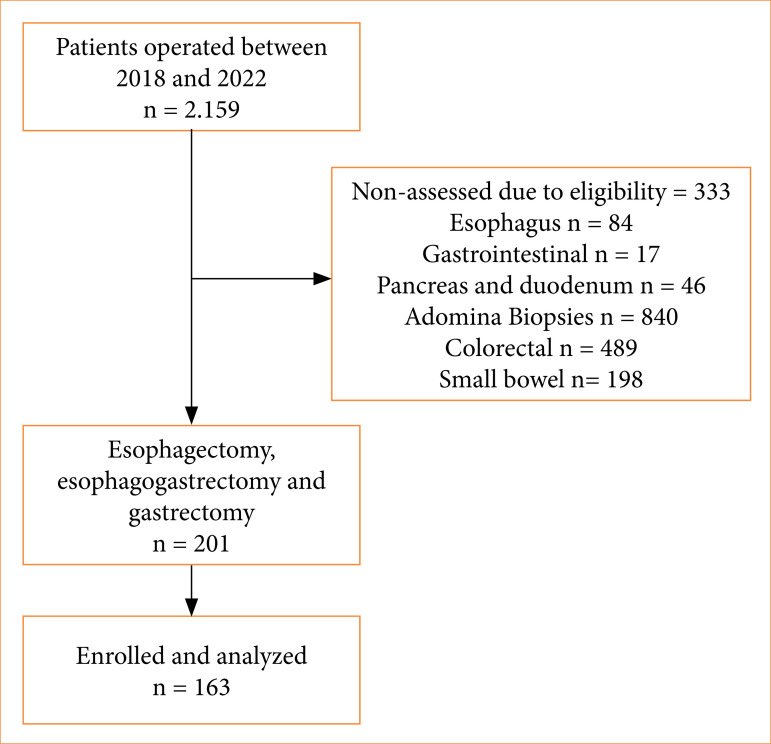
Sample selection flowchart.

Source: Elaborated by the authors.

The total of 2,159 oncologic surgeries were performed between 2018 and 2022, categorized by anatomical sites. Among them, 201 were selected for the esophagus and stomach site, and 144 experienced postoperative complications (Fig. 1).

Table 1 illustrates the demographic and surgical characteristics of the patients in the present study. Among the investigated cases (n = 163), the mean age was 61.8 (standard deviation – SD = 13.3), with the majority being male 94 (57.7%), with mild systemic disease 84 (51.5%), and undergoing open surgical methods 159 (97.5%).

**Table 1:** Demographic and surgical characteristics of patients.

Characteristic	n = 163
**Age**	61.8 (13.3%)
**Gender**	
Female	69 (42.3%)
Male	94 (57.7%)
**ASA score**	
ASA 1: Normal health	47 (28.8%)
ASA 2: Mild systemic disease	84 (51.5%)
ASA 3: Severe systemic disease, not disabling	16 (9.8%)
Not Specified	16 (9.8%)
**Surgical method**	
Open	159 (97.5%)
Laparoscopic	4 (2.5%)
**Surgical Time (minutes)**	
< 180	60 (36.8%)
> 180	103 (63.2%)
**Other sequential procedures**	
Yes	159 (97.5%)
No	4 (2.5%)
**Antibiotic prophylaxis in surgery**	
Yes	162 (99.4%)
No	1 (0.6%)

ASA: American Society of Anesthesiologists. Source: Elaborated by the authors.

The preoperative clinical characteristics were assessed, highlighting that moderate or suspected malnutrition (Subjective Global Assessment – SGA B) and severely malnourished (SGA C) accounted for 38.8 and 14.2% of the sample, respectively (Table 2).

**Table 2:** Clinical characteristics of the patient preoperatively.

Characteristics	Frequency	%
**Nutritional profile**		
Well-nourished or anabolic (SGA A)	63/134	47.0
Moderate malnutrition or suspected malnutrition (SGA B)	52/134	38.8
Severely malnourished (SGA C)	19/134	14.2
**Hypoalbuminemia**	6/54	11.1
**Anemia**	30/119	25.2
**Leukopenia**	10/119	8.4
**Lymphopenia**	9/117	7.7

SGA: Subjective Global Assessment. Source: Elaborated by the authors.

In Table 3, the results regarding characteristics related to identified surgical complications and their classification according to Clavien-Dindo are presented. Thus, 144 patients experienced some postoperative complication classified between Clavien-Dindo I to V, representing an 88.3% overall prevalence. Among them, 81.3% were mild complications, classified between I and II, requiring no major interventions, and 18.7% were more severe complications necessitating more invasive medical interventions, such as procedures, surgical reapproach, and intensive care. The description of complications is depicted in Table 4.

**Table 3:** Characteristics of postoperative complications according to the Clavien-Dindo Classification.

Characteristic	n = 144
Grade I	43 (29.9%)
Grade II	74 (51.4%)
Grade III	1 (0.7%)
Grade IIIa	4 (2.8%)
Grade IIIb	8 (5.5%)
Grade IV	1 (0.7%)
Grade IVb	3 (2.1%)
Grade V	10 (6.9%)

Source: Elaborated by the authors.

**Tabela 4:** Postoperative complications.

Grade I	Grade II	Grade III	Grade IV	Grade V
Pain (5)	Severe anemia (6)	Enteric anastomotic fistulas with peritonitis (3)	Stroke (2)	Sepsis (8)
Mild pulmonar dysfunctions (5)	Anastomotic fistulas (5)	Upper gastrointestinal bleeding (2)	Sepsis (1)	Surgical wound dehiscence with gastroparesis (1)
Patients with no reported complaints (20)	Use of antibiotics for infection (57)	Dehiscence of enteric anastomosis (2)	Acute pulmonar edema (1)	Hepatic cirrhosis (1)
	Parenteral nutrition (3)	Accidental removal of the drain (1)		
		Bradypnea (1)		

Source: Elaborated by the authors.

An association was examined between clinical variables and complications. Among the analysis of variables such as surgical time, surgical method, nutritional profile, hypoalbuminemia, anemia, leukopenia, and lymphopenia, only hypoalbuminemia showed statistical significance (p = 0.0358). Thus, an association was demonstrated between this variable and the occurrence of postoperative complications (Table 5).

**Table 5:** Statistical analysis between variables and the occurrence of complications.

Characteristic	Complication		p-value*
	Yes	No	
**Risk factor**			**0.3466**
ASA 1	9	38	
ASA 2	11	73	
ASA 3	4	12	
Not specified	3	13	
**Surgical time (minutes)**			0.0757
< 180	6	54	
> 180	21	82	
**Surgical method**			0.1283
Open	25	134	
Laparoscopic	2	2	
**Nutritional profile**			0.2996
SGA A	7	56	
SGA B	11	41	
SGA C	2	17	
Not specified	7	22	
**Hypoalbuminemia**			**0.0358**
Yes	3	3	
No	5	43	
Not specified	19	90	
**Anemia**			1.000
Yes	5	25	
No	16	73	
Not specified	6	38	
**Leukopenia**			1.000
Yes	2	8	
No	19	90	
Not specified	6	38	
**Lymphopenia**			0.6329
Yes	2	6	
No	19	90	
Not specified	6	40	

* Fisher’s exact test;

χ^2^ test; ASA: American Society of Anesthesiologists; SGA: Subjective Global Assessment. Source: Elaborated by the authors.

In Clavien-Dindo, death is represented by grade V. Thus, the mortality rate is 6.1% (10) among patients who underwent gastroesophageal oncologic surgery. The logistic regression model was used to evaluate the existence of risk factors associated with severe complications (Grade III and above) in patients. Therefore, based on the information from the Table 6, it can be inferred that patients with hypoalbuminemia are at a higher risk of experiencing complications compared to patients without hypoalbuminemia, with an odds ratio of 8.60 (p = 0.0358).

**Table 6:** Statistical analysis of risk factors associated with grade III and above complications in patients.

Characteristics	OR (95%CI)
**Sex**	
Feminine	1
Masculine	1.93 (0.79–4.72)
**ASA score**	
ASA 1: Healthy patient	1
ASA 2: Mild systemic disease	0.64 (0.24–1.67)
ASA 3: Moderate to severe systemic disease	1.41 (0.37–5.40)
**Surgical method**	
Open	1
Laparoscopic	5.36 (0.72–39.84)
**Surgical time (minutes)**	
< 180	1
> 180	2.30 (0.87–6.08)
**Others sequenced procedures**	
No	1
Yes	3,201,415 (0–Inf)
**Surgical antibiotic prophylaxis**	
No	1
Yes	1,151,562 (0–Inf)
**Nutritional status**	
SGA A	1
SGA B	2.15 (0.77–6.01)
SGA C	0.94 (0.18–4.96)
**Hypoalbuminemia**	
No	1
Yes	8.60 (1.35; 54.64)
**Anemia**	
No	1
Yes	0.91 (0.30–2.75)
**Leukopenia**	
No	1
Yes	1.18 (0.23–6.02)
**Lymphopenia**	
No	1
Yes	1.58 (0.30–8.43)

Anesthesiologists; SGA: Subjective Global Assessment.Source: Elaborated by the authors.

## Discussion

The global prevalence characterized in this study shows that 88.3% of the included patients presented some degree of postoperative complication, with infection being the main one, even with the application of pre- and intraoperative antibiotic prophylaxis, as recommended by the World Health Organization^12^. It was found that more than 50% of the patients were classified as grade II of Clavien-Dindo, having undergone clinical treatment with antibiotic therapy for pulmonary, gastrointestinal, urinary, operative wound, and anastomotic fistula infections.

Among the main complications found in the literature, anastomotic fistula, hemorrhage, abscess, peritonitis, sepsis, and stroke were the most prevalent^13^^–^ 16. In comparison, our results not only demonstrated the prevalence of patients with some form of infection but also anastomotic fistulas, hemorrhages, sepsis, and strokes, consistent with other analyzed studies.

According to the meta-analysis by Pucher et al.^17^, 22.6% of evaluated patients had postoperative complications after tumor resection, and this meta-analysis aligns with the variation between 24.7 and 25.3% found in the literature^13^^,^ 14. Thus, the current study shows a high prevalence of complications in the evaluated period, exposing more than triple the percentage comparatively.

In Galata et al.^13^, the 30-day postoperative mortality rate was 4.7%, a contrast with the 0.8% reported in the study by Yuan et al.^14^. A comparatively mortality rate of 6.1% was found in this study, with septic shock as the leading cause of death.

Corroborating this data, the univariate analysis in the study by Mokart et al.^15^ showed that the occurrence of sepsis postoperatively is a factor that independently increases long-term mortality, a converging result with the main cause of death in this current work.

Furthermore, although the study by Poziomyck et al.^18^ considers the ASA-PS score a reliable parameter in predicting mortality after 30 days of the procedure, considering gastrectomies, in the present article, no statistical match was found between this scale and the occurrence of postoperative complications. In contrast to the variables found in their study, where it was not possible to associate hypoalbuminemia with mortality^18^, our study showed an increased risk of complications in patients with serum albumin levels less than 3.5 g/dL, proving to be a risk factor for more severe complications (Clavien-Dindo greater than 3).

The occurrence of severe postoperative complications is associated with immediate mortality and long-term survival after gastroesophageal surgeries^19^, making the evaluation of complications important for possible prevention of negative outcomes, as well as early identification of risk factors in patients undergoing such procedures to contribute to the decision-making of an appropriate therapeutic plan.

This study has the limitation of data recording in medical records, in which some factors such as the lack of description of the pathology, instituted treatment, and justifications for non-inclusion were not available for inclusion in the analysis.

## Conclusion

Postoperative complications of gastroesophageal surgeries increase patient morbidity and mortality, as described in this study. Accordingly, the prevalence in this study was 88.3%, with a predominance of infections and septic shock as the leading cause of death. Furthermore, a correspondence was established between hypoalbuminemia and a higher occurrence of postoperative complications.

## Data Availability

All data sets were generated or analyzed in the current study.
